# Analysis and Experiments on the Cow-Pock Matter

**Published:** 1803-01-01

**Authors:** 

**Affiliations:** Cassel, and other German Physicians


### *Analysis and Experiments on the Cow-Pock Matter, by Dr.* Hunoldt, *of Cassel, and other German Physicians; communicated by Dr.* Noehden.

1. FRESH cow-pock matter was taken from an entire pustule on the seventh day after inoculation, with a gold needle: it was in a very limpid state. On being applied to a bit of paper tinged with vegetable blue colour, it produced no change on it.

2. A paper, coloured with vegetable blue, being reddened with acetous acid, assumed on a sudden a blue colour, when it was touched over with fresh cow-pock matter.

3. On drying the paper over a coal fire, the blue colour disappeared, and the paper became red again.

4. The blue streaks, which were produced by the cow-pock matter disappeared entirely some days after, notwithstanding the paper was wrapped up so as to prevent the immediate accession of the atmosphere, as much as possible.—Hence it seems to follow,

1. That fresh cow-pock matter is of a subtle and volatile alkaline or ammoniacal nature.

2. That its virus is decomposed or destroyed by a strong degree of heat.

3. That it likewise experiences a decomposition in common temperature, and at the accession of atmospherical air, either by being oxydated through the oxygen of the atmosphere, or by being neutralised with the carbonic acid contained in the atmosphere. From these circumstances it appears, why cow-pock matter ought not to be taken at too late a period, and after it has lost its limpid appearance; why it is in general preferable to take it from a pustule which is still unopened; for the same reason, it ought never to be mixed with water, or exposed to the breath, for fear of destroying the subtle active parts; and it ought, likewise, to be preserved from the accession of the atmosphere. It is, therefore, advisable to make a small wound, and let the dry matter be made liquid by the blood of the wound. These are Dr. Hunoldt's observations. They are opposed by Dr. Bremer, of Berlin, who maintains, that the nature of the cow-pock virus does not consist in its ammonia, as its efficacy seems not to depend on the presence of this constituent. This he endeavours to prove by the following experiments:

1. Matter taken from different species of ulcers and from small-pox pustules, and from those raised by the itch, &c. being applied to reddened paper, changed the red colour immediately into blue, which disappeared on the paper being warmed.

2. Cow-pock matter was taken from genuine cow-pock, on the 13th, 14th, and 16th day after the inoculation; it was of a considerable degree of consistency, and of a yellow colour, and, according to experience, incapable of causing the the genuine cow-pock; however, it produced the same change on the colour of the paper.

3. That sort of cow-pock, which had all the characteristics of the spurious kind, produced a blue colour on the same paper.

4. Dry matter kept on threads in glass tubes, closely shut, and preserved for about one twelvemonth, varied the red colour of the paper into blue, on being moistened with pure water, though it is known that such matter will always prove inefficacious in producing the cow-pock disease. Hence it is manifest, that the nature of this poison is not to be sought for in the volatile ammonia, the latter being mixed with different species of pus and other animal substances, and that cow-pock matter, notwithstanding its being still impregnated with that constituent, may prove ineffectual. It seems also obvious, that if the action of the cow-pock matter originated in its volatile constituent, it would be propagated by the atmosphere, by clothes, by the mere touch, &c. Now it is known, that it is only communicated by means of a wound, therefore that supposition cannot be admitted. It is very probable, that we shall never be able to discover the nature of animal poisons by a chemical analysis; we must, therefore, content ourselves with observing their effects, and finding out, by mere experience, the means of preventing their pernicious effects. Dr. Gautieri, in his account of the progress of the vaccine inoculation in the Cisalpine Republic, remarks, that the cow-pock poison may be preserved in its perfect efficacy for four months, particularly under an idiolectrical receiver: it would appear, as if the matter is decomposed by oxygen and carbonic acid, and that it remains particularly efficacious in very pure azotic gas. It is farther decomposed by light. The analysis does not account for the action of this poison on the living body.

